# Performance of GAAD and GALAD Biomarker Panels for HCC Detection in Patients with MASLD or ALD Cirrhosis

**DOI:** 10.3390/cancers17233835

**Published:** 2025-11-29

**Authors:** Mohammad Jarrah, Sneha Deodhar, Lisa Quirk, Mohammed Al-Hasan, Ashish Sharma, Guruveer Bhamra, Julia Terrell, Fasiha Kanwal, Yujin Hoshida, Nicole E. Rich, Purva Gopal, Amit G. Singal

**Affiliations:** 1 Department of Internal Medicine, UT Southwestern Medical Center, Dallas, TX 75235, USA; 2 Clinical Development and Medical Affairs, Roche Diagnostics International AG, 6343 Rotkreuz, Switzerland; ashish.sharma.as6@roche.com; 3 Department of Internal Medicine, Baylor College of Medicine, Houston, TX 77030, USA; 4 Department of Pathology, UT Southwestern Medical Center, Dallas, TX 75235, USA; 5 Division of Digestive and Liver Diseases, UT Southwestern Medical Center, 5959 Harry Hines Blvd, POB 1, Suite 420, Dallas, TX 75390-8887, USA

**Keywords:** GAAD, GALAD, hepatocellular carcinoma, biomarkers, screening

## Abstract

**Background:** Abdominal ultrasound is prone to hepatocellular carcinoma (HCC) surveillance failure, particularly in patients with metabolic dysfunction-associated steatotic liver disease (MASLD) or alcohol-associated liver disease (ALD), prompting growing interest in blood-based biomarkers as an alternative strategy. **Methods:** We conducted a case–control study evaluating two blood-based biomarker panels, GAAD and GALAD, for detection of early-stage HCC (Barcelona Clinic Liver Cancer (BCLC) stage 0 or A) in patients with MASLD or ALD cirrhosis. Blood specimens were collected within 6 months of HCC diagnosis (cases); controls were patients with cirrhosis but without HCC. GAAD and GALAD scores were measured using the Roche Elecsys platform, applying validated cutoffs of 2.57 and 2.47, respectively. Sensitivity and specificity were compared between the panels and versus ultrasound plus alpha fetoprotein (AFP) using McNemar’s chi square test. **Results:** Of 152 patients (56.6% men), 46.7% had HCC (54.9% BCLC 0/A) and 53.3% had cirrhosis without HCC. GAAD and GALAD each achieved a sensitivity of 87.2% for early-stage HCC, with specificities of 69.1% and 67.9%, respectively. In paired analyses (n = 90), GAAD had higher sensitivity for any-stage HCC (89.5% vs. 68.4%, *p* = 0.046) but lower specificity (71.8% vs. 93.0%, *p* = 0.006) than ultrasound plus AFP. GAAD and GALAD demonstrated consistently higher sensitivity than ultrasound plus AFP across subgroups by age, sex, cirrhosis etiology, and Child Pugh class. **Conclusion:** In this case–control study of patients with non-viral cirrhosis, GAAD and GALAD demonstrated high sensitivity for early-stage HCC. These findings highlight the potential of blood-based biomarkers to improve HCC surveillance in contemporary populations.

## Simple Summary

Hepatocellular carcinoma (HCC) is the most common form of primary liver cancer and is often diagnosed at advanced stages. Detecting HCC early improves survival, but current screening with ultrasound has notable limitations and can miss up to one third of cases, particularly among patients with non-viral liver disease. This study evaluated blood-based biomarker panels, including GAAD and GALAD, and compared their performance with ultrasound for identifying early-stage HCC in contemporary, non-viral liver disease populations. We found that GAAD and GALAD panels showed high sensitivity for detecting early-stage HCC but low specificity. These findings underscore the potential of biomarkers to improve early detection of HCC and increase access to curative treatment and cancer screening. Our results support ongoing efforts to improve HCC screening, including further evaluation of these biomarker panels in validation studies among non-viral liver disease patients.

## 1. Introduction

Hepatocellular carcinoma (HCC) is the third leading cause of cancer-related mortality worldwide [1]. HCC prognosis is stage dependent, with 5-year survival rates exceeding 60% for patients with early-stage tumors, compared to less than 20% for those with advanced disease. Consequently, early detection through routine surveillance is essential for improving patient outcomes. Society guidelines recommend semi-annual abdominal ultrasound plus serum alpha-fetoprotein (AFP) for patients with cirrhosis and those with chronic hepatitis B viral (HBV) infection [2,3]. However, the effectiveness of ultrasound-based surveillance is suboptimal, with a sensitivity of only 63% for early-stage HCC detection, and is further limited by factors such as operator expertise, patient obesity, and underlying liver disease etiology [4,5,6,7,8,9]. The value of surveillance must also consider physical, financial, and psychological harms [10,11]. Finally, suboptimal adherence, stemming from both patient- and provider-level barriers, further limits the effectiveness of surveillance in clinical practice [12,13,14].

These limitations have increased interest in emerging imaging- and blood-based strategies for HCC surveillance. Multi-phase CT and MRI have shown promise as alternative modalities for HCC surveillance [15,16,17]. However, blood-based biomarkers have the potential to improve both sensitivity and adherence, which could lead to marked improvements in overall surveillance effectiveness. Genetic alterations that drive HCC development, including TERT promoter, TP53, VEGF, WNT/β-catenin signaling, and the mTOR pathways, are linked to the expression of potential surveillance biomarkers, such as AFP [18,19,20,21,22]. However, single biomarkers have insufficient accuracy, highlighting the need for multi-biomarker panels. A case–control study demonstrated that AFP plus des-gamma-carboxy prothrombin (DCP) provided the highest accuracy among two-biomarker combinations [23]. Adding a third biomarker (e.g., lens culinaris agglutinin-reactive fraction of AFP (AFP-L3)), along with age and sex, further increased sensitivity for early-stage HCC detection.

Accordingly, commercial biomarker panels based on this combination are available, including GAAD (Elecsys platform), ASAP (ARCHITECT platform), and GALAD (μTASWako platform). Each has demonstrated promising results in phase 2 biomarker studies, with a meta-analysis of existing literature reporting pooled sensitivities between 70% and 74% for early-stage HCC [24,25,26,27,28,29,30,31,32,33,34,35,36]. However, data are limited for contemporary cirrhosis populations, particularly among patients with metabolic dysfunction-associated steatotic liver disease (MASLD), metabolic dysfunction and alcohol-associated liver disease (Met-ALD), and alcohol-associated liver disease (ALD). HCC surveillance remains suboptimal in patients with MASLD and ALD, including lower utilization and worse diagnostic performance for early-stage detection compared to viral etiologies [37,38,39]. Notably, up to one third of MASLD-related HCC occurs in the absence of cirrhosis, further challenging the effectiveness of current surveillance strategies [40,41,42].

Given the known influence of liver disease etiology on biomarker performance, data from these populations are critical [43]. Further, most studies have evaluated biomarker performance in isolation or compared with AFP, with few directly comparing their accuracy against ultrasound plus AFP, the current standard of care. Herein, we conducted a case–control study to evaluate the performance of two blood-based biomarker panels, GAAD and GALAD, in patients with MASLD- or ALD-related cirrhosis.

## 2. Materials and Methods

### 2.1. Study Population

We conducted a retrospective case–control study leveraging a biorepository of serum samples collected between October 2014 and February 2020 from patients at the University of Texas Southwestern Medical Center and Parkland Health, two large health systems in Dallas County. Cases were defined as patients with treatment-naïve HCC, and controls as patients with cirrhosis without evidence of HCC for at least 1 year of follow-up. HCC was diagnosed using the American Association for the Study of Liver Diseases (AASLD) criteria, and cirrhosis was diagnosed by histology, transient elastography, serum fibrosis markers, or cirrhotic-appearing liver with portal hypertension on imaging [3]. All cases and controls had MASLD, ALD, or Met-ALD.

Baseline clinical data included age, sex, race and ethnicity, body mass index (BMI), and cirrhosis etiology and severity (per Child Pugh class). Race and ethnicity were categorized as non-Hispanic White (White), non-Hispanic Black (Black), non-Hispanic Asian (Asian), and Hispanic. Cirrhosis etiology was classified as MASLD, ALD, or Met-ALD; due to small numbers, patients with Met-ALD were combined with ALD group for analysis. For patients with HCC, we recorded tumor characteristics (number of liver lesions, maximum tumor diameter, vascular invasion, distant metastasis, ECOG performance status). Early-stage HCC was defined as Barcelona Clinic Liver Cancer (BCLC) stage 0 or A. The study protocol (STU092013-010) was approved by the Institutional Review Board at UT Southwestern Medical Center.

### 2.2. Sample Processing and Biomarker Measurement

All samples were processed according to Early Detection Research Network protocols, with all specimens processed within 4 h of collection and stored at −80 °C without interim thawing or re-freezing [44]. Samples were shipped to Roche for analysis, where serum levels of AFP, AFP-L3, and DCP were measured using the Roche Elecsys immunoassay platform by staff blinded to HCC status. Abnormal values were defined using pre-specified thresholds: AFP ≥ 20 ng/mL, AFP-L3 > 2.3 ng/mL, and DCP > 28.4 ng/mL. A GALAD score ≥ 2.47 and GAAD score ≥ 2.57 were considered positive, based on manufacturer-established cutoffs [45].

### 2.3. Ultrasound Assessment

Ultrasound results were obtained from the electronic medical record, when available. At both sites, ultrasounds were performed as part of routine care by registered sonographers and interpreted by fellowship-trained radiologists [46]. An abnormal ultrasound was defined as detecting a liver lesion ≥ 1 cm in diameter. When available, ultrasound visualization quality was recorded per LI-RADS criteria: score A for minimal limitations, score B for moderate limitations, and score C for severe limitations [47].

### 2.4. Statistical Analysis

Diagnostic performance for each biomarker panel was initially assessed using the area under the receiver operating characteristic curve (AUROC), with AUROCs compared using DeLong’s test. Sensitivity and specificity for any-stage and early-stage HCC were calculated for each surveillance strategy. Differences in test performance were assessed using McNemar’s chi-square test. For comparisons between biomarkers and ultrasound, we restricted analyses to patients with blood collection within 6 months of ultrasound. Test performance was evaluated overall and across subgroups, including liver disease etiology, Child-Pugh class, and tumor burden. All statistical analyses were performed using R version 4.4.2.

## 3. Results

### 3.1. Patient Characteristics

Characteristics of the 71 cases with HCC and 81 controls with cirrhosis are described in Table 1. Cases were older than controls, with median ages of 64.9 and 55.0 years, respectively. Overall, 56.6% of the patients were men, and 42.0% were non-Hispanic White, with no significant differences between groups. Liver disease etiology was similar between cases and controls, with MASLD accounting for 52.6% and ALD for 47.4%. Half (53.3%) had Child-Pugh class A cirrhosis, with compensated cirrhosis observed in 43.7% of cases and 64.9% of controls. Median BMI was 30.4, with more than half of patients classified as obese. Among cases, 57.7% had a unifocal tumor, with a median tumor diameter of 3.9 cm. Nearly half (46.5%) met Milan Criteria. BCLC staging was distributed as follows: 54.9% stage 0/A, 9.9% stage B, 23.9% stage C, and 11.3% stage D.

### 3.2. Individual Biomarkers

Compared to patients with cirrhosis, those with HCC had higher median values of AFP (31.8 vs. 3.0 ng/mL), AFP-L3 (5.7 vs. 1.2%), and DCP (446.4 vs. 46.6 ng/mL) (*p* < 0.001 for all) (Supplemental Figure S1). For early-stage HCC detection, AUROCs were 0.80 (95% CI 0.71–0.90) for AFP, 0.75 (95% CI 0.67–0.84) for AFP-L3, and 0.77 (95% CI 0.68–0.86) for DCP. AFP sensitivities were 56.3% for any-stage HCC and 51.3% for early-stage HCC, with a specificity of 98.8%. AFP-L3 sensitivities were 57.7% for any-stage and 48.7% for early-stage, with a specificity of 97.5%. DCP demonstrated higher sensitivities of 95.8% for any-stage and 92.3% for early-stage, but a lower specificity of 30.9%.

### 3.3. GAAD and GALAD

**Figure 1:**
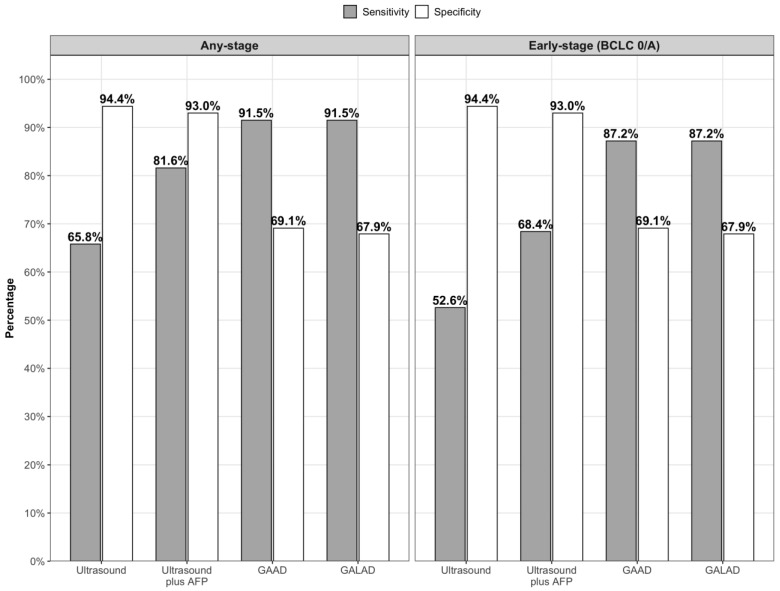
Diagnostic performance of ultrasound, ultrasound plus AFP, GAAD, and GALAD for any-stage and early-stage (BCLC 0/A) HCC.

Compared to patients with cirrhosis, those with HCC had higher median values of GAAD (9.6 vs. 1.3) and GALAD (9.6 vs. 1.4) (*p* < 0.001 for both) (Supplemental Figure S2). Diagnostic accuracy for GAAD and GALAD, is illustrated in Figure 1, and their performance using established cut-offs is shown in Table 2. For any-stage HCC, GAAD had an AUROC of 0.92 (95% CI 0.88–0.97), with 91.5% sensitivity and 69.1% specificity. For early-stage HCC, GAAD had an AUROC 0.90 (95% CI 0.84–0.96), with 87.2% sensitivity. Similarly, GALAD had an AUROC of 0.92 (95% CI 0.88–0.97) for any-stage HCC, with 91.5% sensitivity of and 67.9% specificity. GALAD had an AUROC of 0.90 (95% CI 0.84–0.96) for early-stage HCC, with 87.2% sensitivity. Differences in AUROC between GAAD and GALAD were not statistically significant for any-stage (*p* = 0.96) or early-stage HCC (*p* = 0.39). Performance was also similar across subgroups defined by liver disease etiology and Child-Pugh class.

### 3.4. Abdominal Ultrasound

Ultrasound within 6 months of blood collection was available for 109 patients (38 cases and 71 controls). Most (77.8%) were performed in the outpatient setting; only 7.4% were performed outside UT Southwestern or Parkland Health. Among 99 patients with available visualization scores, 63.6% had score A, 30.3% score B, and 6.1% score C. Ultrasounds with limited visualization (score B or C) were more common among patients with HCC compared to cirrhosis controls (51.4% vs. 27.7%, *p* = 0.04). Ultrasound alone demonstrated sensitivities of 65.8% for any-stage HCC and 52.6% for early-stage HCC, with a specificity of 94.4%. When combined with AFP, sensitivity increased to 81.6% for any-stage and 68.4% for early-stage, with a small decrease in specificity (93.0%).

### 3.5. GAAD vs. Ultrasound Plus AFP

Among patients with early-stage HCC who had biomarker and ultrasound data within 6 months (n = 90), GAAD had significantly higher sensitivity for early-stage HCC than ultrasound plus AFP (89.5% vs. 68.4%; *p* = 0.046) but lower specificity (71.8% vs. 93.0%; *p* = 0.006) (Supplemental Table S1). GAAD had higher sensitivity than ultrasound plus AFP in most subgroups, including age (<65 years: 80.0% vs. 80.0%, *p* = 1.0; ≥65 years: 100% vs. 55.6%, *p* = 0.046), sex (men: 86.7% vs. 66.7%, *p* = 0.08; women: 100% vs. 75.0%, *p* = 0.32), liver disease etiology (MASLD: 87.5% vs. 75.0%, *p* = 0.32; ALD: 90.9% vs. 63.6%, *p* = 0.08), and Child Pugh class (Child Pugh A: 100% vs. 77.8%, *p* = 0.16; Child Pugh B-C: 80.0% vs. 60.0%, *p* = 0.16). GALAD also demonstrated higher sensitivity for early-stage HCC and lower specificity compared to ultrasound plus AFP, with similar patterns observed in subgroup analyses (Supplemental Table S1).

Ultrasound plus GAAD had the same sensitivity for early-stage HCC as GAAD alone (89.5% vs. 89.5%), with lower specificity (69.0%; *p* = 0.16). Ultrasound plus GAAD had significantly higher sensitivity than ultrasound alone (*p* = 0.008) and ultrasound plus AFP (*p* = 0.046), but with significantly lower specificity (*p* < 0.001 for both).

## 4. Discussion

Given ultrasound’s limitations, there has been increasing interest in emerging surveillance modalities such as blood-based biomarkers. In this study, both GAAD and GALAD demonstrated high sensitivity for early-stage HCC detection, with no significant difference between the two panels. Among patients with available ultrasound data, both biomarker panels achieved higher sensitivity than ultrasound plus AFP, with consistent results across most subgroups examined; however, they are limited by low specificity. Overall, our findings highlight the promise of blood-based biomarkers for HCC surveillance and support their continued evaluation in future cohort studies [30].

Ultrasound has been the standard of care for HCC surveillance for over 20 years, yet its limitations for detecting early-stage HCC are increasingly recognized. Test performance is notably worse in patients with obesity and non-viral cirrhosis, partly due to hepatic steatosis, which impairs visualization. Our results align with prior literature demonstrating reduced ultrasound visualization in patients with non-viral etiologies and Child Pugh B cirrhosis, reinforcing the limited effectiveness of ultrasound-based surveillance in these populations [8]. Interestingly, we found ultrasound visualization was more frequently impaired in patients with HCC, further highlighting the importance of alternative strategies. From an imaging perspective, abbreviated MRI has significantly higher sensitivity than ultrasound, including in those with non-viral etiologies [48,49,50,51,52]. While MRI-based surveillance may be appropriate for selected patients, limited radiologic capacity, higher costs, and logistical barriers to adherence make it impractical as a universal solution [53,54]. In contrast, blood-based biomarkers offer practical advantages such as objective interpretation and integration into routine clinic visits, which may improve adherence. Patients undergoing blood-based biomarker surveillance during routine clinic visits may avoid many ultrasound challenges such as scheduling difficulties as well as financial and psychological burdens [10,11]. Decision modeling supports the cost-effectiveness of blood-based biomarker surveillance compared to ultrasound, considering test performance, adherence, and costs [55,56]. Costs of panels will need to be considered as they are further validated and widely available. However, despite these theoretical advantages and favorable test performance, most studies to date are case–control or retrospective cohort designs, underscoring the need for prospective validation. Large-scale trials, such as the National Liver Cancer Screening Trial (TRACER), will be critical in defining the role of blood based-biomarker surveillance strategies [57].

Most prior studies evaluating blood-based biomarkers have been conducted in patients with viral hepatitis, making our study an important addition to the literature by providing data in contemporary MASLD- and ALD-populations [24]. These etiologies are known to reduce the sensitivity of ultrasound and are also associated with lower levels of certain biomarkers, including AFP [39,58]. In our cohort, both GAAD and GALAD demonstrated equivalent clinical performance across subgroups by etiology and Child Pugh class, which is in line with previously published studies [26,33,59].

Furthermore, both GAAD and GALAD achieved significantly higher sensitivity than ultrasound plus AFP, albeit with lower specificity. This trade-off is generally acceptable to both patients and providers, who tend to prioritize sensitivity and potential benefits over specificity and surveillance-related harms [60,61]. Although this lower specificity increases the risk of false positive results and unnecessary investigations, prior studies suggest that the harms of surveillance are limited compared to the benefits of early HCC detection. These patients often undergo additional diagnostic evaluations, including CT or MRI imaging but typically avoid invasive procedures such as liver biopsy [3]. A stakeholder panel of hepatology and cancer screening experts still suggested emerging strategies should maintain a specificity of at least 80% [62], a threshold not reached by either panel in our study. While physical harms from diagnostic follow-up evaluation are generally mild, the psychological and financial harms from false positive results can be clinically significant [10,11]. Indeed, both panels had specificity estimates below 70%, highlighting potential harms in nearly one-third of patients. Future prospective studies should quantify these harms, as harms may be mitigated compared to specificity with biomarker-based strategies. In contrast to our study, recent phase 3 data using a different GALAD cut-off on the μTASWako platform reported adequate sensitivity and specificity [35,63]. Similarly, evaluation of GAAD and GALAD in cohorts with viral liver disease demonstrated high specificity [24]. These data suggest that optimization of cutoff thresholds and platform selection may help balance sensitivity and specificity in non-viral cirrhosis populations. Studies have shown that lowering the cut-off for AFP optimizes the diagnostic performance in patients with MASLD and ALD [39].

Future surveillance strategies may include AI-based methods to optimize biomarker panel selection or weights or incorporate other surveillance modalities to achieve high sensitivity while maintaining adequate specificity [64,65,66,67]. Emerging methylated DNA markers and panels have shown promising performance for early HCC detection [68,69,70,71,72,73,74]. There is also growing evidence supporting the use of using abbreviated MRI, alone or in combination with biomarkers such as AFP, to enhance the overall performance of HCC surveillance [48,75].

We acknowledge several limitations in our study. First, its case–control design is subject to selection and spectrum biases, which may overestimate test performance. Second, our sample size was limited, limiting power for subgroup analyses of interest. Similarly, although our data suggest that GAAD and GALAD had similar performance, aligning with prior data [76,77], our study was not powered for comparisons between the panels. Third, cases and controls differed in age and liver disease severity, which may have impacted biomarker performance. Finally, patients with Met-ALD were combined with the ALD group given small numbers (n = 3), which limited evaluating biomarker performance in Met-ALD as a distinct group. Nonetheless, our study is among the first to directly compare GAAD and GALAD to ultrasound plus AFP in a non-viral cirrhosis population.

## 5. Conclusions

In conclusion, GAAD and GALAD provide higher sensitivity compared to ultrasound plus AFP, but with low specificity. Our findings support the continued evaluation of biomarker panels, both as a complement to, and potential replacement for, ultrasound-based surveillance in patients with non-viral cirrhosis.

## Figures and Tables

**Table 1:** Baseline characteristics of patients with (cases) and without (controls) HCC.

	HCC Cases (n = 71)	Cirrhosis Controls (n = 81)	*p*-Value
Age			<0.001
<65	36 (50.7%)	70 (86.4%)	
≥65	35 (49.3%)	11 (13.6%)	
Sex			0.07
Women	25 (35.2%)	41 (50.6%)	
Men	46 (64.8%)	40 (49.4%)	
Race and Ethnicity			0.32
Hispanic White	37 (52.1%)	39 (48.1%)	
Non-Hispanic White	28 (39.4%)	36 (44.4%)	
Black	4 (5.6%)	1 (1.2%)	
Asian	2 (2.8%)	5 (6.2%)	
Liver disease Etiology			0.87
ALD	33 (46.5%)	39 (48.1%)	
MASLD	38 (53.5%)	42 (51.9%)	
Child-Pugh Class			0.01
A	31 (43.7%)	50 (64.9%)	
B/C	40 (56.3%)	27 (35.1%)	
Body mass index category			0.68
Normal	10 (14.1%)	14 (17.3%)	
Overweight	24 (33.8%)	22 (27.2%)	
Obesity class I	20 (28.2%)	19 (23.5%)	
Obesity class II	9 (12.7%)	16 (19.8%)	
Obesity class III	8 (11.3%)	10 (12.3%)	
Number of HCC tumors, median (IQR)	1 (1–2)	-	-
Maximum tumor diameter (cm), median (IQR)	3.9 (2.6–6.8)	-	-
BCLC staging		-	-
Stage 0	3 (4.2%)		
Stage A	36 (50.7%)		
Stage B	7 (9.9%)		
Stage C	17 (23.9%)		
Stage D	8 (11.3%)		

Continuous variables are expressed as median (p25–p75) BCLC—Barcelona Clinic Liver Cancer; ALD—Alcohol associated liver disease; MASLD—metabolic dysfunction associated steatotic liver disease.

**Table 2:** Diagnostic performance of biomarkers for any-stage and early-stage (BCLC 0/A) HCC detection.

Test	AUROC	Sensitivity	Specificity
**Any-stage HCC (n = 71 cases, 81 controls)**			
Ultrasound (n = 109)	0.80 (95% CI 0.72–0.88)	65.8% (95% CI 48.6–80.4%)	94.4% (95% CI 86.2–98.4%)
Ultrasound plus AFP (n = 109)	0.88 (95% CI 0.80–0.96)	81.6% (95% CI 65.7–92.3%)	93.0% (95% CI 84.3–97.7%)
GAAD (n = 152)	0.92 (95% CI 0.88–0.97)	91.5% (95% CI 82.5–96.8%)	69.1% (95% CI 57.9–78.9%)
GALAD (n = 152)	0.92 (95% CI 0.88–0.97)	91.5% (95% CI 82.5–96.8%)	67.9% (95% CI 56.6–77.8%)
**Early-stage HCC (n = 39 cases, 81 controls)**			
Ultrasound (n = 90)	0.73 (95% CI 0.62–0.85)	52.6% (95% CI 28.9–75.6%)	94.4% (95% CI 86.2–98.4%)
Ultrasound plus AFP (n = 90)	0.79 (95% CI 0.64–0.93)	68.4% (95% CI 43.4–87.4%)	93.0% (95% CI 84.3–97.7%)
GAAD (n = 120)	0.90 (95% CI 0.84–0.96)	87.2% (95% CI 72.6–95.7%)	69.1% (95% CI 57.9–78.9%)
GALAD (n = 120)	0.90 (95% CI 0.84–0.96)	87.2% (95% CI 72.6–95.7%)	67.9% (95% CI 56.6–77.8%)

## Data Availability

Data supporting the findings of this study are available within the article and its Supplementary Materials. Other study materials and data related to the study are available from the corresponding author, upon reasonable request.

## Supplementary Materials

The following supporting information can be downloaded at: https://www.mdpi.com/article/10.3390/cancers17233835/s1, Figure S1: Box and whisker plots of individual biomarkers in patients with cirrhosis vs. HCC; Figure S2: Box and whisker plots of biomarker panels in patients with cirrhosis vs. HCC; Table S1: Performance of GAAD and GALAD vs. ultrasound plus AFP for early-stage (BCLC 0/A) HCC detection.
