# Supplementary material for: Performance of GAAD and GALAD Biomarker Panels for HCC Detection in Patients with MASLD or ALD Cirrhosis
Source: Cancers (Basel). 2025 Nov 29;17(23):3835. doi: 10.3390/cancers17233835 (PMC12691257; doi:10.3390/cancers17233835)
Supplement: Supplementary file 1 [file cancers-17-03835-s001.zip › cancers-3978064-supplementary.pdf]

cancers

Supplementary Materials

Performance of GAAD and GALAD Biomarker Panels for HCC Detection in Patients with MASLD or ALD Cirrhosis

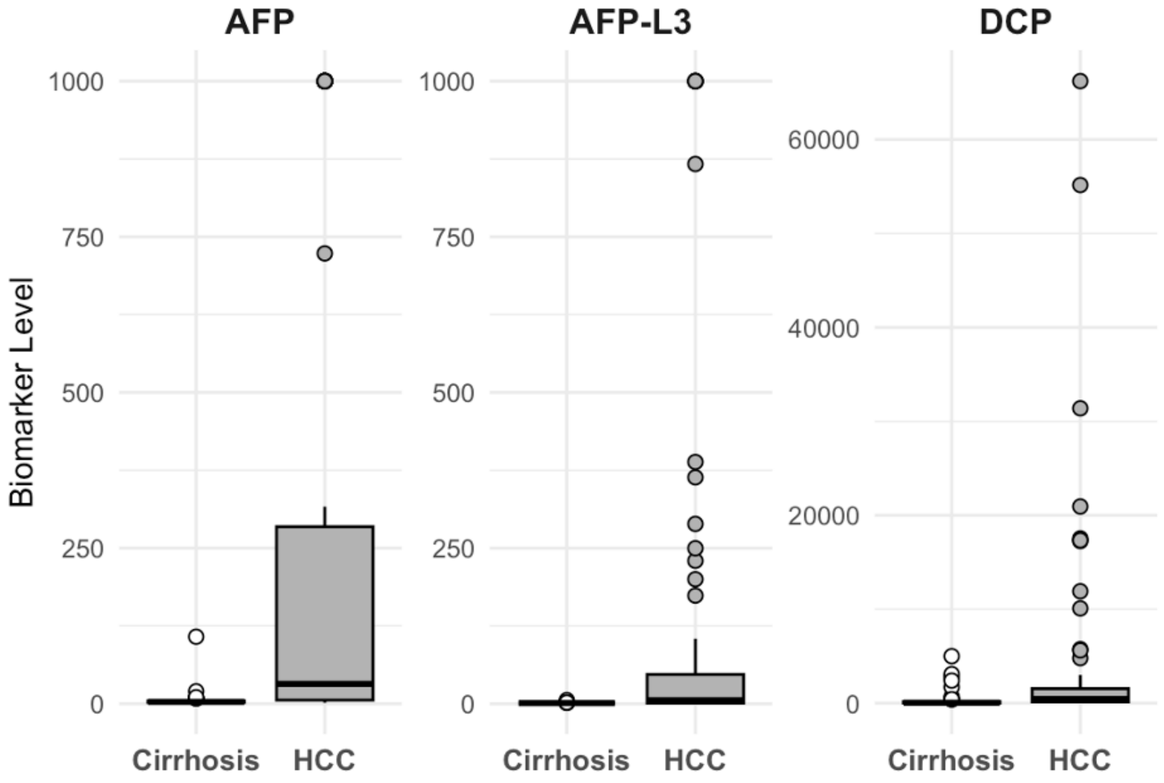

Figure S1. Box and whisker plots of individual biomarkers in patients with cirrhosis vs. HCC.

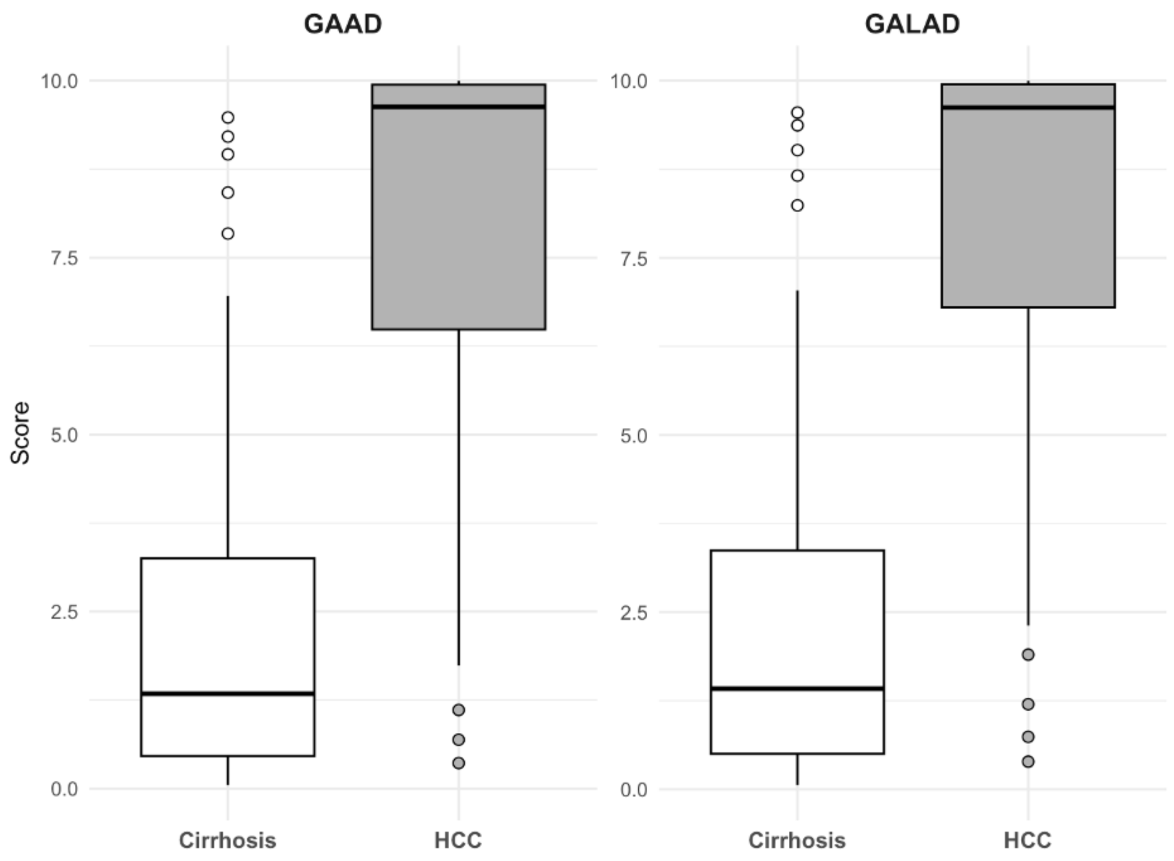

Figure S2. Box and whisker plots of biomarker panels in patients with cirrhosis vs. HCC.

Table S1. Performance of GAAD and GALAD vs. ultrasound plus AFP for early-stage (BCLC 0/A) HCC detection.

Test	GAAD	GALAD	Ultrasound plus AFP	p-value of GAAD vs. ultrasound/AFP	p-value of GALAD vs. ultrasound/AFP
Overall cohort (n = 90)*					
Sensitivity	89.5%	89.5%	68.4%	**0.046**	**0.046**
Specificity	71.8%	70.4%	93.0%	**0.006**	**<0.001**
Diagnostic odds ratio	21.7	20.2	28.6	>0.05	>0.05
Subgroup: age <65 (n = 74)					
Sensitivity	80.0%	80.0%	80.0%	NA	NA
Specificity	70.3%	68.8%	92.2%	**0.001**	**<0.001**
Subgroup: age ≥ 65 (n = 16)					
Sensitivity	100.0%	100.0%	55.6%	**0.046**	**0.046**
Specificity	85.7%	85.7%	100.0%	0.32	0.32
Subgroup: men (n = 48)					
Sensitivity	86.7%	86.7%	66.7%	0.08	0.08
Specificity	54.5%	51.5%	87.9%	**0.005**	**0.003**
Subgroup: women (n = 42)					
Sensitivity	100.0%	100.0%	75.0%	0.32	0.32
Specificity	86.8%	86.8%	97.4%	**0.046**	**0.046**
Subgroup: MASLD (n = 43)					
Sensitivity	87.5%	87.5%	75.0%	0.32	0.32
Specificity	82.9%	80.0%	100.0%	**0.01**	**0.008**
Subgroup: ALD (n = 47)					
Sensitivity	90.9%	90.9%	63.6%	0.08	0.08

Specificity	61.1%	61.1%	86.1%	**0.01**	**0.01**
Subgroup: Child-Pugh A (n = 54)					
Sensitivity	100.0%	100.0%	77.8%	0.16	0.16
Specificity	86.7%	86.7%	91.1%	0.41	0.41
Subgroup: Child-Pugh B/C (n = 32)					
Sensitivity	80.0%	80.0%	60.0%	0.16	0.16
Specificity	45.5%	40.9%	95.5%	**<0.001**	**<0.001**

* Analyses restricted to subset of patients with GAAD and ultrasound available within 6 months of each other.
